# The Relationship between Social Support and Exercise Adherence among Chinese College Students during the COVID-19 Pandemic: The Mediating Effects of Subjective Exercise Experience and Commitment

**DOI:** 10.3390/ijerph191811827

**Published:** 2022-09-19

**Authors:** Yuge Tian, Zhenguo Shi

**Affiliations:** School of Physical Education, Shandong University, Jinan 250061, China

**Keywords:** COVID-19, social support, subjective exercise experience, exercise commitment, exercise adherence, chain mediating effect, Chinese college students

## Abstract

This study aimed to investigate the relationship between social support and exercise adherence among Chinese college students during the COVID-19 pandemic, as well as the mediating effects of subjective exercise experience and commitment in the relationship, in order to provide a reference for improving exercise adherence among Chinese college students during the COVID-19 pandemic. The Perceived Available Support in Sport Questionnaire, Subjective Exercise Experience Scale, Exercise Commitment Scale, and Exercise Adherence Scale were administered to 459 eligible college students in Jinan, Shandong Province, through convenience sampling. Results showed that social support positively predicted exercise adherence among college students. The separate and joint mediating effects of subjective exercise experience and commitment between social support and college students’ exercise adherence were significant. This study demonstrated that social support can positively predict exercise adherence among college students. In addition, subjective exercise experience and commitment played mediation roles between social support and college students’ exercise adherence, both separately and jointly. Therefore, enhancing social support and improving the level of subjective exercise experience and exercise commitment among college students may be an effective strategy for improving exercise adherence among Chinese college students during the COVID-19 pandemic.

## 1. Introduction

Since the emergence of COVID-19, many countries, including the Chinese government, have implemented various levels of prevention and restriction measures, such as containment and isolation, to slow or stop its spread. However, due to the COVID-19 pandemic and its related control measures, the exercise behavior of college students has been negatively affected in many ways [1]. Studies have found that the average duration of physical exercise among Chinese adolescents decreased sharply from 540 min/week to 105 min/week during the pandemic, compared to the pre-pandemic period of COVID-19 [1]. This public health event is still spreading globally, which can have lasting effects on the psychological state and behavioral choices of college students to adhere to exercise [2], and may lead to weight gain, decreased cardiorespiratory fitness [3], depression [4], anxiety [5], and other physical and mental health problems. The COVID-19 pandemic and resulting lack of exercise adherence is an unprecedented health crisis and challenge for college students. Therefore, it is necessary to investigate the factors and potential mechanisms influencing exercise adherence among college students during the COVID-19 pandemic, in order to improve the exercise adherence of college students.

Exercise adherence is a persistent behavioral tendency exhibited by individuals during physical exercise [6]. The lack of exercise adherence among college students during the COVID-19 pandemic may be related to a lack of social support [7]. Social support refers to the respect, care, and help from important people around or other groups close to the individual in an individual’s social network [8]. This support includes emotional support, informational support, and instrumental support for the individual [9]. Previous studies have shown that social support can reduce isolation, suppress mental fatigue, and increase exercise intention among college students during exercise [10,11,12]. Trost et al. [13] found that the perceived social support of college students was related to their exercise adherence. One study also showed that women with high levels of social support were approximately twice as likely to exercise for at least 30 min for five or more days a week as women with low levels of social support [14]. Thus, it is not difficult to speculate that more social support during the COVID-19 pandemic may have contributed to increased exercise adherence among college students.

Previous research has found that exercise behavior is influenced not only by rational decision-making processes, such as cognitive decisions, but also by irrational factors [15]. Among them, subjective exercise experience is one of the important influencing factors [16]. Subjective exercise experience is a subjective emotional memory or feeling formed by an individual during a pre-existing exercise session [17]. Several studies have shown that the positive emotional experiences of exercise reinforces the individuals’ intention to adhere to exercise and makes individuals more inclined to do so [18]. A negative emotional experience of exercise will prevent individuals from forming the intention to persist in exercise and lead to interruption of exercise [19]. On the other hand, subjective exercise experience may also be influenced by social support. Social support can alleviate individuals’ negative emotions through moral and material help from family and friends and make them feel a positive subjective experience [20]. The above studies suggest that subjective exercise experience may mediate the relationship between social support and exercise adherence.

Exercise commitment may mediate the relationship between social support and exercise adherence. Exercise commitment refers to the psychological state of physical exercisers’ desire and determination to engage in physical activity, which is reflected in psychological attachment to physical exercise and behavioral intention to persist in exercise [21]. Many scholars consider exercise commitment as an important indicator and prerequisite for the persistence and stability of exercise behavior [22,23,24]. Han and Yang [25] also showed that exercise commitment is positively associated with exercise adherence; the higher the level of exercise commitment of exercise participants, the stronger their positive perceptions of exercise adherence, which ultimately enhances the likelihood that individuals will engage in exercise adherence. In addition, it was found that social constraints can reduce exercise commitment, while social support can increase the level of exercise commitment of individuals [21,26].

Many studies have suggested that subjective exercise experience may be related to exercise commitment. Social learning theory suggests that the experience of feeling in a particular situation can have an impact on psycho-cognitive decision making [27]. The subjective exercise experience, as a state of fluency, is the basis for an individual’s decision to firmly commit to exercise [28]. Exercisers with poor exercise experience often feel psychologically fatigued [29] and repulsed by the act of exercising, thus resulting in a reduced intention to exercise and wavering commitment to exercise [18]. However, positive exercise experiences lead college students to feel a sense of pleasure and satisfaction, and this experience from a positive source may lead to a clearer intention to exercise and stronger commitment to exercise [16], thus allowing them to maintain a consistent pattern of exercise behavior.

**Figure 1:**
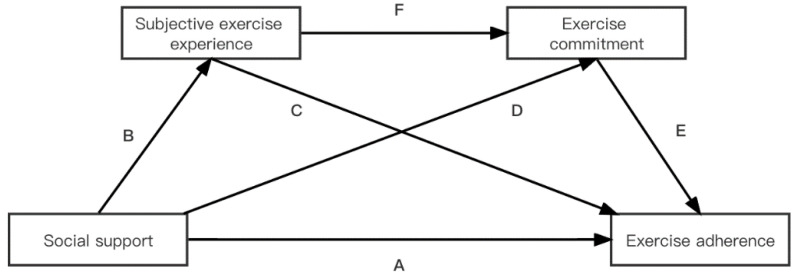
The hypothesis model. Hypothesis 1: A; hypothesis 2: B and C; hypothesis 3: D and E; hypothesis 4: B, F, and E.

The prevalent lack of exercise adherence among Chinese college students during the COVID-19 pandemic has had a serious impact on their physical and mental health. In the context of the normalization of epidemic prevention and control in China, understanding the key factors and mechanisms affecting exercise adherence among Chinese college students could help them cope with the possible adverse psychological and physical problems caused during the COVID-19 pandemic. Although some studies have shown that social support is positively associated with exercise adherence among college students [10,11,12,13], in the context of the COVID-19 pandemic, to our knowledge, no studies have revisited this relationship. In addition, most previous studies have only explored the relationship between the two, and few studies have explored the mediating factors between the two, which is not conducive to a deeper understanding of the relationship and guidance for college students’ exercise adherence in practice. Based on this, this study aimed to investigate: (1) the positive predictive relationship of social support on exercise adherence among college students during the COVID-19 pandemic; (2) the multiple mediating effects of subjective exercise experience and commitment between social support and exercise adherence. The research hypothesis and hypothesis model (Figure 1) proposed for the study are as follows:

**Hypothesis** **1:** *Social support positively predicts college students’ exercise adherence*.

**Hypothesis** **2:** *Subjective exercise experience mediates the relationship between social support and exercise adherence among college students*.

**Hypothesis** **3:** *Exercise commitment mediates the relationship between social support and exercise adherence among college students.*

**Hypothesis** **4:** *Subjective exercise experience and commitment mediate the chain between social support and exercise adherence.*

## 2. Materials and Methods

### 2.1. Participants and Procedures

A convenience sampling method was used to select college students enrolled in several universities in Jinan, Shandong Province, during the COVID-19 pandemic, and questionnaires were distributed to them through the platform of Questionnaire Star (http:www.wjx.cn, (accessed on 1 November 2020). We recruited participants in the following ways: (1) disseminating the questionnaire through social media; (2) identifying a teacher at each school as a contact person and using the teacher’s dissemination to get students to scan the questionnaire link and fill out the questionnaire. A total of 488 enrolled college students eventually participated in the questionnaire, and all participants who took part in the survey gave their informed consent to the study. The Institutional Review Board of the School of Shandong Institute of Sports approved this study. After excluding invalid questionnaires, such as missing answers and insufficient time for testing, 459 valid questionnaires were finally recovered, with a recovery efficiency of 94.06%. At this point, the sample size met the statistical requirement of being at least 5–10 times the number of scale items [30]. The age of the subjects ranged from 19 to 23 (20.85 ± 1.32) years. Among them, 278 (60.57%) were male, and 181 (39.43%) were female; 128 (27.89%) were freshmen, 60 (13.07%) were sophomores, 164 (35.73%) were juniors, and 107 (23.31%) were seniors. In addition, 173 (37.69%) were liberal arts students; 142 (30.94%) were engineering students; and 144 (31.37%) were medical students.

### 2.2. Instruments

For the latent variables in the hypothetical model, we measured them using Perceived Available Support in Sport Questionnaire, Subjective Exercise Experience Scale, Exercise Commitment Scale, and Exercise Adherence Scale.

#### 2.2.1. Perceived Available Support in Sport Questionnaire

The Perceived Available Support in Sport Questionnaire, developed by Freeman et al. [31], was used in this study to measure participants’ social support. The scale contains 4 dimensions, i.e., emotional support, respectful support, informational support, and tangible support, with 10 entries. The scale was scored on a 5-point Likert scale, with a score of 1 for very non-conforming and 5 for very conforming, with higher scores indicating stronger social support for the participants.

#### 2.2.2. Subjective Exercise Experience Scale

The Subjective Exercise Experience Scale, developed by McAuley and Courneya [17], was used in this study to measure participants’ subjective exercise experience. The scale contains two dimensions, positive well-being and psychological distress, with eight entries, and was scored on a scale of 1 to 5, with 1 point for complete noncompliance and 5 points for complete compliance. The higher the score, the higher the subjective exercise experience.

#### 2.2.3. Exercise Commitment Scale

The Exercise Commitment Scale, developed by Scanlan et al. [32] and simplified for Chinese adolescents by Chen, Liu, and Yan [21], was used to assess participants’ level of commitment to exercise adherence. The scale has two dimensions, psychological attachment to physical exercise and behavioral intention, with four items. The scale was scored on a scale of 1 to 5, with a score of 1 for strongly disagree and 5 for strongly agree, with higher scores indicating a stronger level of commitment to exercise.

#### 2.2.4. Exercise Adherence Scale

The Exercise Adherence Scale, developed by Wang, Liu, and Gu [6], was used to measure participants’ exercise adherence. The scale consists of 14 items and contains three dimensions: action habits, effort, and emotional experience. The scale was scored on a scale of 1 to 5, i.e., from 1 to 5 for “completely disagree to completely agree”. The higher the score, the greater the exercise adherence.

### 2.3. Statistical Analyses

This study used self-report method to collect data, so it may bring the problem of common method deviation, so Harman one-way test was used to test the common method deviation after data collection [33].

SPSS 26.0 (IBM, Armonk, NY, USA) was used to perform descriptive and correlation analyses on the data of this study. In addition, considering that the sociodemographic variables of gender and grade may differ significantly among the variables, independent samples *t*-test and one-way ANOVA were used to test for differences in gender and grade among the variables, and both were included as control variables in the subsequent analysis.

Multiple regression analysis was conducted using Model 6 in the SPSS PROCESS macro program (version 3.5), prepared by Hayes. The bias-corrected percentile bootstrap method (5000 replicate samples) was selected to estimate 95% confidence intervals (CI) for the testing of mediation effects [34]. If the 95% confidence interval of the mediating effect did not contain 0, it means that the mediating effect was significant. According to the Wen and Ye [35] steps of the mediation effect test, the mediating effects of subjective exercise experience and commitment in social support and exercise adherence was analyzed, controlling for gender and grade.

## 3. Results

### 3.1. Common Method Deviation Test

The results of the unrotated principal component factor analysis showed that the first factor variance explained 20.260%, which was less than the critical criterion of 40%, indicating that the common method deviation was within acceptable limits. Therefore, there is no serious common deviation in the survey data of this study.

### 3.2. Descriptive Statistical and Correlation Analysis

The means and standard deviations of each variable on the gender and grade dimensions are shown in Table 1. The results of independent samples *t*-test showed that there were significant differences in the scores of subjective exercise experience (*t* = 2.899, *p* < 0.01), commitment (*t* = 4.934, *p* < 0.001), and adherence (*t* = 2.501, *p* < 0.05) among college students of different genders. The results of one-way ANOVA showed that college students of different grades had significantly different scores on social support (*F* = 10.538, *p* < 0.001) and exercise commitment (*F* = 2.685, *p* < 0.05) among college students of different grades. Therefore, we controlled for gender and grade in the following mediation analyses.

As shown in Table 2, Pearson correlation analysis was performed on social support, subjective exercise experience, exercise commitment, and exercise adherence. The results showed that social support was significantly and positively correlated with subjective exercise experience (r = 0.124, *p* < 0.01), a significant positive correlation with exercise commitment (r = 0.280, *p* < 0.01), and a significant positive correlation with exercise adherence (r = 0.378, *p* < 0.01). Exercise adherence was significantly and positively correlated with subjective exercise experience (r = 0.574, *p* < 0.01), and it was significantly and positively correlated with exercise commitment (r = 0.613, *p* < 0.01). Subjective exercise experience was significantly and positively correlated with exercise commitment (r = 0.478, *p* < 0.01). There was a significant two-by-two correlation between the study variables, thus satisfying the statistical requirements for conducting a mediating effects test.

### 3.3. Mediation Effect Analysis

**Figure 2:**
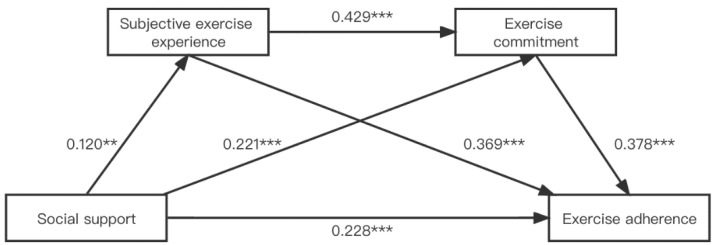
Mediation effect analysis of subjective exercise experience and commitment between social support and exercise adherence; *** *p* < 0.001, ** *p* < 0.01.

The results of the regression analysis (Table 3 and Figure 2) showed that social support was a significant positive predictor of subjective exercise experience (*β* = 0.120, *p* < 0.01). After including social support and subjective exercise experience as independent variables and exercise commitment as dependent variables in the equation, social support (*β* = 0.221, *p* < 0.001) and subjective exercise experience (*β* = 0.429, *p* < 0.001) both significantly and positively predicted exercise commitment. After including social support, subjective exercise experience and commitment as independent variables and exercise adherence as dependent variables in the equation, social support (*β* = 0.228, *p* < 0.001), subjective exercise experience (*β* = 0.369, *p* < 0.001), and exercise commitment (*β* = 0.378, *p* < 0.001) all significantly and positively predicted exercise adherence.

The results of the mediated effect analysis (Table 4) showed that subjective exercise experience and commitment partially mediated the effect between social support and exercise adherence, with a mediated effect value of 0.169, accounting for 39.260% of the total effect. The indirect effect of social support on exercise adherence was mediated through three mediating paths. Mediated path 1 was “social support—subjective exercise experience—exercise adherence”, with an effect value of 0.051. Mediated path 2 was “social support—exercise commitment—exercise adherence”, with an effect value of 0.096. Mediated path 3 was “social support—subjective exercise experience—exercise commitment—exercise adherence”, with an effect value of 0.022. The mediated effects of each path accounted for 11.752%, 22.341%, and 5.167% of the total effects, respectively. The bootstrap 95% confidence interval of the above mediating effects did not contain 0, thus indicating that all three mediating effects reached a significant level; that is, subjective exercise experience and commitment can not only play a mediating effect between social support and exercise adherence alone, but it also plays a chain mediating effect, through “subjective exercise experience—exercise commitment”. In addition, a two-by-two comparison of the mediating effects of different paths was conducted to test whether there was a significant difference between the mediating effects of different paths. The results showed that the bootstrap 95% confidence interval of the difference between mediating effects 1 and 2 (C1) contained 0, thus indicating that there was no significant difference between mediating effects 1 and 2. Bootstrap 95% confidence interval of the difference between mediating effects 1 and 3 (C2) and the difference between mediating effects 2 and 3 (C3) did not contain 0, thus indicating that both sets of mediated effects, compared above, are statistically significant.

## 4. Discussion

### 4.1. The Direct Effect of Social Support on College Students’ Exercise Adherence

The findings of this study confirmed hypothesis 1, in which social support was a significant and positive predictor of exercise adherence among college students. This is consistent with the results of previous studies [36,37] and, again, validates that social support is an important factor predicting exercise adherence. Ecosystem theory suggests that the development of an individual is nested in a systemic environment that interacts with each other, and that the system and individual interact with each other and drive the individual’s continuous development, thus emphasizing the importance of the external environment to the individual’s behavior [38]. In addition, self-determination theory also suggests that support from the external environment can increase the individual’s level of autonomous motivation, which, in turn, promotes individual behavioral persistence [17]. For college students, on the one hand, the perceived autonomy support can fully mobilize the initiative and motivation of exercise, alleviate the hesitation in exercise, and make it possible to improve the persistence of exercise [39]. On the other hand, this social support from the external environment also provides college students with a protective effect against the inhibition of their positive psychology and tendency to persist in their exercise behavior [40]. In addition, a point worth noting is that we found that sophomores scored relatively high on social support, compared to students in other grades. This may be related to the stage of study that sophomores are in. Compared to juniors and seniors, who are under pressure to find a job or continue their studies, and freshmen, who are new to college life, sophomores have more time and opportunities to engage in physical exercise, and, therefore, feel more social support.

### 4.2. The Mediating Effects of Subjective Exercise Experience and Commitment between Social Support and College Students’ Exercise Adherence

The findings of this study confirmed hypothesis 2, that subjective exercise experience mediated the relationship between social support and exercise adherence among college students. The results support the previous findings that social support is positively related to subjective exercise experience. The results support the previous findings that social support is positively correlated with subjective exercise experience [41], and subjective exercise experience further positively predicts exercise adherence among college students [18,19]. Social support provides college students with objective tangible support and provides them with subjective emotional care, which strengthens their perception and awareness of positive exercise experience and strongly influences their future exercise behavior [40]. Drive theory suggests that an individual’s internal drive, due to a state of deprivation and the experience of effects gained from observation and experience, regulate the level of effort and persistence of an individual in performing an activity or behavior [42]. This further indicates that positive subjective exercise experiencers will develop regular physical exercise, while negative subjective exercise experiencers will resist or even quit physical exercise. Therefore, in the strategy of promoting college students’ exercise adherence, focusing on making college students gain more fluency and satisfaction in exercise, is an effective means to avoid them from quitting exercise in the middle.

In addition, this study also found that exercise commitment mediated the relationship between social support and exercise adherence among college students, and hypothesis 3 held true. Previous research has shown that, in the absence of internal motivation to exercise, perceived social support by college students reinforces their intrinsic motivation, which, in turn, triggers higher levels of exercise commitment [43]. Higher levels of exercise commitment also imply higher levels of exercise attachment and intention to exercise, which map to individual college students’ beliefs regarding their will to stay physically active. In addition, studies have shown that psychological commitment actually reflects the stability and continuity of a relationship and plays a key effect in adherence behavior [44]. As a psychological commitment, in the exercise context, exercise commitment also has an important influence on college students’ exercise adherence. Providing college students with good external support and enhancing their level of exercise commitment is an important guarantee to support their exercise adherence.

Finally, the findings of this study also confirmed hypothesis 4, i.e., that subjective exercise experience and commitment mediated the chain between social support and exercise adherence. Subjective exercise experience can have a direct predictive effect on exercise commitment, as suggested by the dual-system processing model of decision making: rational psychological factors cannot be reinforced and activated by irrational factors on the subject’s decision making [45]. College students with high levels of perceived social support always have positive and enjoyable exercise experiences or experiences and can perceive the efficacy of physical activity for self-actualization and self-improvement, thus making it easier to maintain the desire and determination to continue exercising [46], which ultimately enables college students to maintain long-term stable and regular exercise behavior.

### 4.3. Implications

This study had the following advantages. (1) The data were primary data obtained through questionnaire collection. (2) The instruments used in this study were a validated and proven scale, and the relationship between social support, subjective exercise experience, exercise commitment, and exercise adherence among college students was further explored with the help of the instruments. In the context of the COVID-19 pandemic, this study explores the exercise adherence of college students, as well as its relevant factors and potential mechanisms, with important theoretical and practical implications. At the theoretical level, this study clarifies the important effect of social support in enhancing exercise adherence among college students, in the context of the COVID-19 pandemic, a global public health event, which actually enriches and expands the ecosystem theory. In addition, this study also confirmed the chain mediating effect of subjective exercise experience and commitment between social support and college students’ exercise adherence, which helps us to understand the potential mechanisms of action between social support and college students’ exercise adherence. At the practical level, firstly, multiple parties, including parents, teachers, and schools, should pay attention to the lack of exercise adherence of college students during the COVID-19 pandemic, which seriously affects their physical and mental health, and provide them with objective material help and subjective emotional support in time to enhance their exercise adherence. Specific measures to increase and develop this social support include the frequent use of affirmative forms of praise-based assessment for students [47] and frequent group counseling sessions that focus on enhancing social support. Secondly, the intervention efficiency of college students’ exercise adherence can be improved by enhancing their subjective exercise experience and strengthening their exercise commitment level through the deployment of new exercise equipment, as well as the introduction of VR somatosensory technology.

### 4.4. Limitations and Future Research Directions

In addition, there are some limitations and shortcomings in this study. First, the study used a cross-sectional design, and the causal relationships among the variables need to be further verified through longitudinal follow-up and experimental design. Second, this study only considered the mediating effects of subjective exercise experience and commitment; however, there may, in fact, be other mediating variables, such as autonomous motivation, exercise climate, and self-efficacy, that need to be further explored. Third, the number of male participants in this study was higher. This should be noted in the interpretation. Fourth, the questionnaire distribution in this study was conducted through the internet only. Follow-up studies should attempt to incorporate offline questionnaire distribution. Fifth, this study focuses on the important role of social support on exercise commitment and exercise adherence. However, the importance is only one aspect, and future research based on this study should pay attention to more in-depth and extended research on how to increase and develop social support. Sixth, the results of this study were derived in the context of the COVID-19 pandemic, which should be noted in the interpretation. Whether the results of this study can continue to be applied beyond the end of the COVID-19 pandemic will require further studies in the future to confirm. Finally, the study used self-reported measures of the variables, so bias may be introduced, and more objective indicators need to be included to measure the study variables in the future.

## 5. Conclusions

This study explored the relationship between social support and exercise adherence among college students during the COVID-19 pandemic and its internal mechanisms of action through a chain mediation model. The study showed that there were significant differences in the scores of social support, subjective exercise experience, exercise commitment, and exercise adherence among college students by gender and grade level. The study also showed that social support had a significant positive predictive effect on exercise adherence among college students. In addition, subjective exercise experience and commitment partially mediated the effect between social support and exercise adherence, which consisted of three pathways: the separate mediation of subjective exercise experience and commitment and chain mediation through subjective exercise experience and commitment. The strongest mediating effect of the three mediated pathways was produced by exercise commitment separately. This shows that social support, subjective exercise experience, and exercise commitment are all key factors predicting exercise adherence among college students and have a positive impact on exercise participation and the development of exercise habits during the COVID-19 pandemic.

## Figures and Tables

**Table 1:** Descriptive statistical results and analysis of variance of the sample.

Variables	Category	Statistical Values	Social Support	Subjective Exercise Experience	Exercise Commitment	Exercise Adherence
Gender	Male	M±SD	61.90 ± 6.020	29.19 ± 5.133	14.36 ± 3.071	52.75 ± 7.773
	Female	M±SD	61.37 ± 7.676	27.74 ± 5.383	12.90 ± 3.165	50.92 ± 7.473
		t	0.830	2.899	4.934	2.501
		p	0.407	0.004	0.000	0.013
Grade	Freshman	M±SD	60.23 ± 6.520	28.91 ± 5.185	14.05 ± 3.149	52.42 ± 7.690
	Sophomore	M±SD	65.80 ± 4.108	28.08 ± 4.311	14.12 ± 2.387	53.37 ± 5.915
	Junior	M±SD	61.79 ± 6.832	29.13 ± 5.213	13.95 ± 3.137	51.86 ± 7.622
	Senior	M±SD	60.97 ± 7.092	27.78 ± 5.873	13.03 ± 3.594	51.07 ± 8.623
		F	10.538	1.772	2.685	1.291
		p	0.000	0.152	0.046	0.277

Note: The numbers in Table 1 represent the mean and standard deviation of each dimension; *t*-test results and significance for each dimension on gender; one-way ANOVA results and significance for each dimension on grade.

**Table 2:** Correlation analysis for each variable.

Variables	***M* ± *SD***	1	2	3	4
1 Social support	37.96 ± 6.014	1			
2 Subjective exercise experience	28.62 ± 5.275	0.124 **	1		
3 Exercise commitment	13.78 ± 3.186	0.280 **	0.478 **	1	
4 Exercise adherence	52.03 ± 7.700	0.378 **	0.574 **	0.613 **	1

Note: ** *p* < 0.01.

**Table 3:** Regression analysis of the relationship between variables in the chain mediation model.

**Variables**	**Subjective Exercise Experience**			**Exercise Commitment**			**Exercise Adherence**		
	β	*SE*	t	β	*SE*	t	β	*SE*	t
Gender	−0.125	0.516	−2.614 **	−0.147	0.266	−3.580 ***	0.034	0.539	1.002
Grade	−0.017	0.224	−0.358	−0.045	0.115	−1.117	−0.027	0.229	−0.793
Social support	0.120	0.036	2.589 **	0.221	0.019	5.611 ***	0.228	0.039	6.763 ***
Subjective exercise experience				0.429	0.024	10.777 ***	0.369	0.054	10.017 ***
Exercise commitment							0.378	0.094	9.771 ***
$R^{2}$	0.033			0.305			0.528		
F	5.093 **			49.719 ***			101.200 ***		

Note: ** *p* < 0.01, *** *p* < 0.001.

**Table 4:** Significance test results for differences in intermediate effects and effect values.

Effect	Standardized Coefficient	Bootstrap SE	Bootstrap 95% CI Lower Limit	Bootstrap 95% CI Upper Limit	Relative Effect Share (%)
Total indirect effect	0.169	0.041	0.092	0.250	39.260
SS → SEE → EA	0.051	0.024	0.008	0.100	11.752
SS → EC → EA	0.096	0.020	0.057	0.137	22.341
SS → SEE → EC → EA	0.022	0.011	0.003	0.045	5.167
C1	−0.046	0.030	−0.100	0.016	
C2	0.028	0.015	0.004	0.061	
C3	0.074	0.022	0.030	0.115	

SS, social support; SEE, subjective exercise experience; EC, exercise commitment; EA, exercise adherence; C1, the effect of the “SS → SEE → EA” minus the effect of the “SS → EC → EA”; C2, the effect of the “SS → SEE → EA” minus the effect of the “SS → SEE → EC → EA”; C3, the effect of the “SS → EC → EA” minus the effect of the “SS → SEE → EC → EA”.

## Data Availability

The data presented are available on request from the corresponding author.
